# Prevalence and Knowledge Levels of Polycystic Ovarian Syndrome among Female Adolescents in Educational Institutions in Hyderabad, India: A Cross-sectional Study

**DOI:** 10.4314/ejhs.v34i6.12

**Published:** 2024-11

**Authors:** Jahnavi Nallavothu, Harshitha Thimmapathini, Adithi Janagama, Durdana Fathima, Meera Indracanti

**Affiliations:** 1 Department of Anesthesia & OTT, School of Allied Health Sciences, Malla Reddy University, Hyderabad, India; 2 Department of Anesthesia & OTT, School of Allied Health Sciences, Malla Reddy University, Hyderabad, India; 3 Department of Anesthesia & OTT, School of Allied Health Sciences, Malla Reddy University, Hyderabad, India; 4 Department of Anesthesia & OTT, School of Allied Health Sciences, Malla Reddy University, Hyderabad, India; 5 Department of Medical Biotechnology, School of Allied Health Sciences, Malla Reddy University, Hyderabad, India

**Keywords:** Awareness, Female Adolescents, Hyderabad, India, Knowledge, PCOS, Prevalence

## Abstract

**Background:**

Polycystic ovarian syndrome (PCOS) is a complex endocrine condition that is frequently misdiagnosed, and its prevalence is rising rapidly worldwide. Adolescent girls often lack adequate knowledge about PCOS. This study examined the prevalence of PCOS and awareness among female adolescents.

**Method:**

A cross-sectional study was conducted among female adolescents from selected educational institutions in the north zone of Hyderabad. Institutions were carefully chosen to represent a diverse student population. Data were collected and analyzed using IBM SPSS Statistics version 25. The Chi-square test of independence and logistic regression were employed to identify variables associated with PCOS knowledge levels.

**Results:**

Three hundred eighty-two female adolescents participated. The mean age was 18.19 years (±0.92 SD). Forty-four participants were diagnosed with PCOS (11.5%), and 17.3% were suspected PCOS. Over 89% of participants demonstrated good knowledge across various PCOS domains. A significant association was found between the level of education and learning about PCOS (p=0.05). There was a weak positive correlation between the level of study and knowledge scores (p=0.05). Bivariate and multivariate logistic analysis revealed that university girls [AOR: 1.9, 95% CI: (1.12-3.25)] and those with PCOS [AOR: 6.8, 95% CI: (1.4-32.4)] were more knowledgeable than their counterparts with lower education levels and without PCOS, respectively.

**Conclusion:**

While knowledge of PCOS among female adolescents was generally good, the disease burden was high. Targeted counseling and lifestyle management interventions are essential to prevent long-term complications of PCOS.

## Introduction

Polycystic ovarian syndrome (PCOS) is a common and complex endocrine condition characterized by an overproduction of androgens. This often leads to misdiagnosis due to its heterogeneous presentation. Common features of PCOS include ovarian cysts, oligo- or anovulation, and hyperandrogenism, all of which adversely affect fertility and reproductive health in women of childbearing age. Globally, PCOS affects approximately 6% to 10% of women of reproductive age, with varying prevalence across different ethnicities and regions.

The incidence of PCOS is rising among adolescents, coinciding with increases in obesity, diabetes, and metabolic syndrome. This condition contributes to lifelong health complications, including metabolic and cardiovascular disorders. PCOS currently affects over 116 million (3.4%) women worldwide. Studies have projected a global prevalence of PCOS between 2.2% and 48% in 2020, highlighting significant geographical variations. Research indicates that 80% of PCOS patients are overweight, while 20% fall within the normal weight range. In India, the prevalence of PCOS ranges from 3.7% to 22.5%, with higher frequencies observed in urban areas compared to rural regions.

Despite the increasing prevalence of PCOS, particularly among adolescents, many females lack adequate knowledge about this syndrome. There is limited data available in India regarding the prevalence and awareness of PCOS among female adolescents. This knowledge gap is concerning, as early identification and management of PCOS can significantly improve long-term outcomes and quality of life. The number of adolescent and young women with PCOS is growing, but the underlying causes remain unclear. Increased awareness is crucial for facilitating earlier detection, better symptom management, and reduced associated health risks.

Few studies demonstrate low awareness of PCOS among adolescents and young women. The present study aims to fill this gap by determining the prevalence of PCOS and examining the factors associated with general awareness, symptoms, and risk factors among female adolescents in selected educational institutions in the northern zone of Hyderabad, Telangana, India. By focusing on this specific population, we hope to emphasize the importance of education and early intervention in mitigating the health burden of PCOS among young females.

## Methods

**Study design, area, source, and participants**: An institutional-based cross-sectional study was conducted from March to May 2023 to assess PCOS prevalence and knowledge levels among adolescent girls in selected educational institutions in the north zone of Hyderabad, Telangana, India. The study was conducted within a single institution comprising several schools and colleges, with most respondents from the college-level student population.

**Inclusion and exclusion criteria**: Female adolescents aged 13 to 19 who were willing to participate and had received verbal or written consent from parents were included in the study. Girls younger than 13 or older than 19, as well as those who did not provide consent, were excluded.

**Sample size**: The sample size was calculated using a finite population formula and Scalex SP. The expected prevalence was 10% (based on a previous study), with a 95% confidence interval and a margin of error of ±3. The required sample size was determined to be 382. A non-response rate was not factored into this calculation, as measures were in place to ensure a minimum response rate to meet the sample size requirement.

**Questionnaire development**: A pretested 38-item knowledge questionnaire and a 30-item PCOS prevalence questionnaire were used. The prevalence questionnaire was based on the validated Rotterdam criteria and included both open- and closed-ended (multiple choice) questions in English. The questionnaire was developed based on study objectives, literature review, and a pilot study to ensure reliability and comprehensibility. Prior to the main study, 5% of the sample size was piloted, and those responses were not included in the final dataset as the pilot aimed to refine the study design and methodology.

**Study variables**: Variables included age, place of birth, level of study, area of study, parents' educational qualifications and occupations, prevalence of PCOS, general awareness, symptoms, and risk factors.

**Sampling and data collection procedure**: The study included 382 female adolescents aged 13–19 from selected educational institutions. Participants were chosen through random sampling and multistage recruitment. Trained laboratory staff collected data from the selected participants.

**Statistical analysis**: All variables of interest were summarized using descriptive statistics. Age, mean, and standard deviation were calculated for continuous variables. Prevalence was assessed using a 30-item instrument, with identifiers scoring 1 for “regular” and 0 otherwise. The maximum score was 30, divided into three categories (Table 1).

**Table 1:** Ranges of prevalence score on PCOS for assessment

*PCOS Prevalence Levels	Score
Normal adolescent girls	0-10
Girls at the Edge of PCOS	11-15
PCOS affected girls	>16

* PCOS category

The knowledge levels of female adolescents regarding symptoms and risk factors were measured using a 38-item instrument. A score of 1 was assigned for a correct answer and 0 for an incorrect answer or “Do not know.” The maximum possible score was 38, divided into two categories (Table 2).

**Table 2:** Ranges of knowledge scores on various broad domains on PCOS for assessment

Knowledge Levels	Score
Low knowledge	≤19
Good knowledge	>19

The impact and strength of variables on knowledge levels regarding various broad domains of PCOS were investigated using the Chi-square test of independence, Pearson's correlation, and binary and multivariate logistic regression.

**Ethical considerations**: The study and manuscript preparation adhered to ethical guidelines, ensuring confidentiality throughout the process.

## Results

**Socio-demographic characteristics of female adolescents**: The study involved 382 female adolescents with a 100% response rate (Table 3). The study participants were 13 to 19 years old, with a mean age of 18.19 (±0.92SD). More than fifty percent of girls were from urban areas (N=202; 52.9%), and most were from Telangana. Only 6.8% of the study participants' fathers' (N=26) and 10.7% of mothers' educational Level (N=41) were illiterate. More than 77% of the students' fathers had some occupation or business, whereas 76.2% of the mothers were homemakers. Forty-four girls were diagnosed with PCOS (11.5%).

**Table 3:** Demographics of female adolescent students, N=382

Demographics of female adolescents		N	%
*Level of Education*			
School		28	7.3
Junior college		198	51.8
University		156	40.8
*Area of Study*			
Healthcare students		213	55.8
Non-Healthcare students		137	35.9
Others		32	8.4
*Father's Education*			
Illiterate		26	6.8
Primary		53	13.9
Secondary		103	27.0
Higher Secondary		102	26.7
University		98	25.7
*Mother's Education*			
Illiterate		41	10.7
Primary		75	19.6
Secondary		116	30.4
Higher Secondary		69	18.1
University		81	21.2
*Father's occupation*			
Employed		158	41.4
Business		138	36.1
Unemployed		9	2.4
Other		77	20.2
*Mother's occupation*			
Employed	62		16.2
Business	22		5.8
Homemaker	291		76.2
Others	7		1.8
*BMI Category*			
Underweight	144		37.7
Healthy weight	169		44.2
Overweight	51		13.4
Obese	18		4.7
*PCOS Category*			
Normal	272		71.2
Edge (Suspected)	66		17.3
PCOS affected	44		11.5

The mean age of the respondents is 18.19 (±0.92SD)

**Knowledge levels of female adolescents on various broad categories of PCOS (N=382)**: Table 4 presents the responses of adolescent students regarding various domains of PCOS. More than 89% of the girls were familiar with at least one aspect of PCOS. However, only 72.9% of the girls exhibited good knowledge across the domains (N=223). Furthermore, only 63% of the participants understood the risk factors associated with PCOS (N=243).

**Table 4:** Knowledge score on broad categories of female adolescents, N=382

Knowledge score on PCOS	N	%
***General Awareness***		
Low knowledge	114	29.8
Good knowledge	268	70.2
***Knowledge on symptoms***		
Low knowledge	134	35.1
Good knowledge	248	64.9
***Knowledge of risk factors***		
Low knowledge	139	36.4
Good knowledge	243	63.6
***Total knowledge score***		
Low knowledge	119	31.2
Good knowledge	263	68.8

**Chi^2^ Test of Independence analysis of knowledge levels of female adolescents on various broad domains of PCOS**: Chi-square analysis showed a significant association between the level of education, as well as the educational backgrounds of both fathers and mothers, with the knowledge levels across various domains of PCOS (p≤0.05) (Table 5).

**Table 5:** Chi^2^ test of Independence on general awareness, symptoms, risk factors, and total knowledge score on PCOS among female adolescents, N=382

Knowledge score of the female adolescents	Chi^2^ value	P value
***Level of Education***		
General Awareness	13.9	0.001
Knowledge on Symptoms	6.66	0.036
Knowledge of Risk Factors	10.77	0.005
Total knowledge of PCOS	8.3	0.016
***Father's Education***		
General Awareness	14.5	0.006
Total knowledge of PCOS	15.4	0.004
***Mother's Education***		
General Awareness	14.1	0.007
Knowledge of Risk Factors	13.3	0.010
Total knowledge of PCOS	16.8	0.002
***PCOS Category***		
General Awareness	12.1	0.002
Knowledge on Symptoms	17.4	0.000
Knowledge of Risk Factors	18.8	0.000
Total knowledge of PCOS	23.2	0.000

**Spearman's correlation analysis**: There was a weak positive correlation between the level of education, the father's education, and the PCOS category concerning knowledge scores across various domains (P≤0.05) (Table 6).

**Table 6:** Correlation of knowledge score of female adolescents on various broad domains, N=382

Knowledge Score of the female adolescents	Pearson's r value	P value (2-tailed)
***Level of Education***		
General Awareness	0.191	0.001
Knowledge of risk factors	0.162	0.002
Total knowledge of PCOS	0.146	0.004
***Father's Education***		
General Awareness	0.159	0.002

Knowledge on Symptoms	0.108	0.035
Knowledge of Risk Factors	0.108	0.035
Total knowledge of PCOS	0.175	0.001
***Mother's Education***		
General Awareness	0.112	0.028
Knowledge of Risk Factors	0.119	0.019
Total knowledge of PCOS	0.161	0.002
***PCOS Category***		
General Awareness	0.175	0.001
Knowledge on Symptoms	0.184	0.001
Knowledge of Risk Factors	0.222	0.001
Total knowledge of PCOS	0.247	0.001

**Factors associated with PCOS knowledge among female adolescents (N=382)**: Bivariate and multivariate logistic regression analysis indicated that the level of education and the PCOS category were significantly associated with knowledge levels across various domains of PCOS (Table 7). University students demonstrated greater knowledge compared to their peers in lower educational levels. Additionally, girls diagnosed with PCOS had significantly higher knowledge levels, being 14 times more knowledgeable [AOR: 14.0, 95% CI: (3.2-60.0)] than their non-PCOS counterparts. Similarly, those on the edge of PCOS were 6.8 times more knowledgeable [AOR: 6.8, 95% CI: (1.4-32.4)].

**Table 7:** Multiple logistic regression of knowledge score of female adolescents on various broad domains and PCOS category, P=0.05; N=382

Category	Knowledge		COR (95%CI)	AOR (95%CI)
**Level of Education**	***Low knowledge***	***Good knowledge***		
***General Awareness***				
School	13	15	0.284(0.12-0.673)	3.9(1.6-9.5)
Junior College	70	128	0.497(0.3-0.8)	2.2(1.2-3.8)
University	31	125	Reference	Reference
***Knowledge on Symptoms***				
School	16	12	0.359(0.157-0.82)	4.6(1.8-11.7)
Junior College	68	130	0.581(0.36-0.93)	#1.3(0.8-2.2)
University	50	106	Reference	Reference
***Knowledge of Risk factors***				
School	16	12	0.295(0.12-0.67)	4.08(1.6-10)
Junior College	79	119	0.592(0.37-0.92)	1.67(1.0-2.7)
University	44	112	Reference	Reference
***Total knowledge of PCOS***				
School	13	15	0.258(0.102-0.65)	3.8(1.5-9.7)
Junior College	69	129	0.523(0.307-0.89)	1.9(1.12-3.25)
University	37	119	Reference	Reference

**PCOS Category**	***Low knowledge***	***Good knowledge***		

***General Awareness***				
Normal	95	177	0.297(0.119-0.74)	3.4(1.3-8.5)
Edge (Suspected)	13	53	#0.570(0.195-1.6)	#1.79(0.6-5.2)
PCOS affected	6	38	Reference	Reference
***Knowledge on Symptoms***				
Normal	106	166	0.115(0.035-0.37)	10.9(3.1-37)
Edge (Suspected)	25	41	0.120(0.034-0.42)	10.9(2.9-40)
PCOS affected	3	44	Reference	Reference
***Knowledge of Risk factors***				
Normal	116	156	0.161(0.06-0.43)	6.2(2.3-16.6)
Edge (Suspected)	18	48	0.289(0.096-0.86)	3.4(1.15-10.4)
PCOS affected	5	39	Reference	Reference
***Total knowledge of PCOS***				
Normal	103	169	0.078(0.019-0.33)	14.0(3.2-60.4)
Edge (Suspected)	14	52	0.177(0.038-0.82)	6.8(1.4-32.4)
PCOS affected	2	42	Reference	Reference

# Not significant

## Discussion

Data on the prevalence of PCOS in India is limited. This study is the first to compare knowledge levels among female adolescents with and without PCOS in the region. Our study found that the prevalence of PCOS among adolescent girls was 11.5%. This is consistent with a similar study conducted in Hyderabad, which reported a prevalence of 11.96% among teenage girls aged 15-19 (13). However, it is lower than the 22.5% prevalence reported in Maharashtra (15) and 28.5% in Dallas (16). Our findings were also higher than the rates observed in South India (9.13%) (17) and Bhopal (8.34%) (18).

We identified that 17.3% of adolescent females were suspected to have PCOS, which is lower than the 32.93% reported in a 2022 study (14). These discrepancies in prevalence rates may stem from differences in study demographics, lifestyle factors, and environmental influences. Factors such as early puberty, rapid fetal development, family history of PCOS, lack of physical activity, stress, and obesity may contribute to variations in prevalence (19). Additionally, differing diagnostic criteria can affect detection rates (20).

Our study found that most female adolescents with PCOS were 18 years old, with an average age of menarche at 13. This differs from another study that reported an average age of 16 and menarche at 12(21).

In this study, 89% of adolescent girls demonstrated good knowledge across various domains of PCOS. In contrast, a survey conducted in Malaysia found that only 41% of teenage girls had a solid understanding of the condition (14). Our study also indicated that only 63% of participants had good knowledge of PCOS risk factors. Prior studies have shown that factors such as educational level, field of study, and personal history of PCOS diagnosis significantly influence awareness of the condition (14).

There was a notable association between the level of education and the knowledge levels of various categories of PCOS (P=0.05). A similar study indicated a significant correlation between awareness and factors such as prior diagnosis, personal connections with affected individuals, work in healthcare, and information sourced from medical professionals (22). Our findings showed a weak positive correlation between education level and knowledge scores (P≤0.05).

Bivariate and multivariate logistic analysis revealed that university students [AOR: 1.9, 95% CI: (1.12-3.25)] and those diagnosed with PCOS were more knowledgeable [AOR: 6.8, 95% CI: (1.4-32.4)] compared to students with lower education levels and those without PCOS.

The results suggest that familiarity with PCOS is closely linked to personal experiences with the condition, as the participants' understanding was largely influenced by their backgrounds (22). Surveys indicate that over 50% of individuals with PCOS consider themselves knowledgeable about the condition (23). Approximately 66% of informed participants had personal connections to someone diagnosed with PCOS, while 20% reported being diagnosed themselves (22).

This analysis highlights the relationship between educational backgrounds and knowledge of PCOS, suggesting that education significantly influences understanding of the condition among the study population. Those with higher education levels tend to have a better grasp of PCOS compared to those with lower educational backgrounds. This underscores the importance of educational interventions to enhance awareness and knowledge about PCOS, particularly for those with lower educational attainment.

Recognizing the impact of education on knowledge levels about PCOS can help healthcare providers, policymakers, and educators design targeted interventions. These initiatives can bridge knowledge gaps and promote informed decision-making regarding PCOS management and treatment. They can guide the development of educational programs aimed at improving awareness, early detection, and support for individuals from diverse educational backgrounds.

The findings indicate that educational attainment and the presence of PCOS are associated with increased knowledge levels among adolescent girls. This highlights the crucial role of education and personal experience in shaping awareness regarding PCOS. The significant results from the logistic analysis reinforce the need for effective strategies to improve awareness, early detection, and support for individuals affected by this condition.

The strength of this study lies in its assessment of PCOS knowledge levels and prevalence. It may facilitate early identification of PCOS in adolescent girls, leading to timely intervention. The cross-sectional design enabled a one-time data collection that illuminates PCOS prevalence and knowledge among female adolescents. Our statistical analysis quantifies awareness levels and prevalence rates. The study emphasizes the need for education on PCOS management to enhance reproductive health among female adolescents. Moreover, it addresses a specific demographic gap in research, focusing on a group of educational institutions under a single organization in the northern zone of Hyderabad. Its findings may apply to similar populations and inform the development of targeted interventions and screening programs.

However, the present study has limitations, including sample size and selection bias. Certain groups may be overrepresented or underrepresented, which could affect the validity of the study. Additionally, our research was confined to a specific group of educational institutions due to time and resource constraints, limiting the breadth of data collected. Participants' responses may also suffer from self-reporting bias. The cross-sectional nature of the study restricts the ability to identify causal connections, offering only a snapshot of prevalence and knowledge levels. The knowledge assessment tool may not capture the full complexity of understanding or nuances of PCOS among adolescent girls. Respondents may provide socially desirable answers rather than accurate ones, influencing perceived knowledge levels and prevalence rates.

Educational programs, screening plans, and support services can aid adolescents with PCOS in obtaining early identification, education, and support, ultimately enhancing their health and quality of life. Awareness and screening programs can facilitate early detection, allowing for timely interventions. Support services can address both emotional and practical needs, improving overall quality of life and mental well-being. Education regarding lifestyle changes and treatment options can help alleviate symptoms.

In conclusion, this study provides critical insights into the prevalence and knowledge of PCOS among female adolescents, with an overall positive knowledge score. The prevalence of PCOS among adolescent girls was high. The study advocates for improved awareness, early identification, counseling, and lifestyle modifications to mitigate the emotional impact of PCOS on adolescent girls and enhance their reproductive health.
